# Development and validation of a Modified Patient‐Generated Subjective Global Assessment as a nutritional assessment tool in cancer patients

**DOI:** 10.1002/jcsm.12872

**Published:** 2021-12-04

**Authors:** Zhenming Fu, Rui Zhang, Kun‐Hua Wang, Ming‐Hua Cong, Tao Li, Min Weng, Zeng‐Qing Guo, Zeng‐Ning Li, Zhao‐Ping Li, Chang Wang, Hong‐Xia Xu, Chun‐Hua Song, Cheng‐Le Zhuang, Qi Zhang, Wei Li, Han‐Ping Shi, The INSCOC Study Group

**Affiliations:** ^1^ Cancer Center Renmin Hospital of Wuhan University Wuhan China; ^2^ Department of Surgery The First Affiliated Hospital, Kunming Medical University Kunming China; ^3^ Department of Comprehensive Oncology National Cancer Center/National Clinical Research Center for Cancer/Cancer Hospital, Chinese Academy of Medical Sciences and Peking Union Medical College Beijing China; ^4^ Department of Radiotherapy, Affiliated Cancer Hospital, School of Medicine UESTC Chengdu China; ^5^ Department of Medical Oncology Fujian Provincial Cancer Hospital, Fujian Medical University Cancer Hospital Fuzhou China; ^6^ Department of Nutrition The First Hospital, Hebei Medical University Shijiazhuang China; ^7^ Center for Human Nutrition David Geffen School of Medicine at UCLA Los Angeles CA USA; ^8^ Cancer Center The First Hospital, Jilin University Changchun China; ^9^ Department of Clinical Nutrition Daping Hospital, Third Military Medical University (Army Medical University) Chongqing China; ^10^ Department of Epidemiology College of Public Health, Zhengzhou University Zhengzhou China; ^11^ Department of Gastrointestinal Surgery Shanghai Tenth People's Hospital, Tongji University Shanghai China; ^12^ Department of Gastrointestinal Surgery, Department of Clinical Nutrition Beijing Shijitan Hospital, Capital Medical University Beijing China; ^13^ Department of Oncology Capital Medical University Beijing China; ^14^ Beijing International Science and Technology Cooperation Base for Cancer Metabolism and Nutrition Beijing China

**Keywords:** Patient‐generated subjective global assessment, PG‐SGA, mPG‐SGA, Nutritional assessment tool, Cancer patient

## Abstract

**Background:**

Completing Patient‐Generated Subjective Global Assessment (PG‐SGA) questionnaires is time consuming. This study aimed to develop and validate an easy‐to‐use modified PG‐SGA (mPG‐SGA) for cancer patients.

**Methods:**

Seventy professionals assessed the content validity, comprehensibility, and difficulty of the full PG‐SGA. A survey including the PG‐SGA and other questionnaires was completed by 34 071 adult hospitalized cancer patients with first cancer diagnosis or recurrent disease with any tumour comorbidities from the INSCOC study. Among them, 1558 patients were followed for 5 years after admission. Reliability and rank correlation were estimated to assess the consistency between PG‐SGA items and to select mPG‐SGA items. The external and internal validity, test–retest reliability, and predictive validity were tested for the mPG‐SGA via comparison with both the PG‐SGA and abridged PG‐SGA (abPG‐SGA).

**Results:**

After deleting items that more than 50% of professionals considered difficult to evaluate (Worksheet 4) and items with an item‐total correlation <0.1, the mPG‐SGA was constructed. Nutritional status was categorized using mPG‐SGA scores as well‐nourished (0 points) or mildly (1–2 points), moderately (3–6 points), or severely malnourished (≥7 points) based on the area under curve (0.962, 0.989, and 0.985) and maximal sensitivity (0.924, 0.918, and 0.945) and specificity (1.000, 1.000, and 0.938) of the cut‐off scores. The external and internal validity and test–retest reliability were good. Significant median overall survival differences were found among nutritional status groups categorized by the mPG‐SGA: 24, 18, 14, and 10 months for well‐nourished, mildly malnourished, moderately malnourished, and severely malnourished, respectively (all *P*s < 0.05). Neither the PG‐SGA nor the abridged PG‐SGA could discriminate the median overall survival differences between the well‐nourished and mildly malnourished groups.

**Conclusions:**

We systematically developed and validated the mPG‐SGA as an easier‐to‐use nutritional assessment tool for cancer patients. The mPG‐SGA appears to have better predictive validity for survival than the PG‐SGA and abridged PG‐SGA.

## Introduction

Malnutrition is prevalent among cancer patients, with a reported incidence ranging from 39% to 87%.^1^ Malnutrition not only reduces the efficacy of antitumour treatment and increases the length of hospital stay but also reduces the quality of life and survival of patients.^2^, ^3^, ^4^, ^5^ Nutritional screening is a process to fast detect whether a patient at risk of malnutrition or not, whereas nutritional assessment finally determines nutritional status. Currently, there is no gold standard for assessing the nutritional status of cancer patients, although various nutritional assessment tools have been developed.^6^ The Patient‐Generated Subjective Global Assessment (PG‐SGA), a modified version of the Subjective Global Assessment (SGA), is the most widely used tool for assessing the nutritional status of cancer patients^2^, ^3^, ^4^, ^5^ because it shows better sensitivity, specificity, positive, and negative predictive values than other tools.^1^ However, it has been considered overly time consuming in China and other countries.^2^, ^7^, ^8^ In addition, some items of the PG‐SGA are perceived as hard to comprehend even by well‐trained professionals.^9^

The abridged PG‐SGA (abPG‐SGA) is a modified version of the PG‐SGA that uses only the patient‐generated component (Boxes 1–4) to provide a simplified tool for nutritional assessment.^10^, ^12^ Moreover, the abPG‐SGA is used as a nutritional screening tool rather than a nutritional assessment tool.^11^, ^12^ Therefore, there is a need to develop a simple validated version of the PG‐SGA to be used as a nutritional assessment tool for cancer patients. The Chinese Society of Nutritional Oncology (CSNO) took on this initiative to develop a modified version of the PG‐SGA without compromising its validity.^10^

In this study, we first surveyed clinical nutrition practitioners about their perception and use of the PG‐SGA and requested advice regarding how to modify it. We then further selected individual items by rigorous statistical analyses. Subsequently, we generated and validated the modified PG‐SGA (mPG‐SGA) as a nutritional assessment tool for cancer patients.

## Materials and methods

### Population

The Investigation on the Nutritional Status and Clinical Outcomes of Common Cancers (INSCOC) is an ongoing national survey conducted in China by the CSNO.^13^ The present study was part of the INSCOC and was conducted between May 2013 and April 2019 in 72 tertiary hospitals across China. Participants were older than 18 years with a pathologically confirmed diagnosis of cancer and were conscious, were able to communicate in Chinese, were willing to participate in this study and provided written informed consent. Participants included patients with first cancer diagnosis or recurrent disease with any tumour comorbidities. The exclusion criteria were those who underwent organ transplantation, pregnant women, or those who were admitted to the intensive care unit (ICU) at the beginning of recruitment. All admitted patients were interviewed by professionals to complete formatted questionnaires including the PG‐SGA, Nutritional Risk Screening 2002 (NRS 2002), Eastern Cooperative Oncology Group (ECOG) performance status (PS), Karnofsky performance status (KPS), and others. Anthropometric measurements were performed by trained medical professionals. The anthropometric measurement included height, body weight, body mass index (BMI), upper midpoint arm circumference, triceps skin fold thickness (non‐dominant arm), and hand grip strength (non‐dominant hand). Laboratory tests were performed immediately after admission by the clinical labs of the participating hospitals, including haemoglobin, albumin, total protein, and C‐reaction protein. All professionals involved in the nutritional status assessment are certified nutritional therapists (registered dietitian/doctors/nurses) with nutrition assessment qualifications and experiences. A total of 41 117 patients were initially included in the study. Patients with incomplete questionnaires (*n* = 4 669) or no data for haemoglobin or albumin (*n* = 2377) were excluded from the study. The current analyses included 34 071 patients. Among them, 1558 of the patients were followed up for 5 years after admission.

### Patient‐Generated Subjective Global Assessment score

The PG‐SGA score is made up of the scores of seven separate domains. The domains are about weight loss (Box 1, Worksheet 1), food intake (Box 2), symptoms (Box 3), activities and functions (Box 4), disease (Worksheet 2), metabolic demand (worksheet 3), and the results of a physical examination (Worksheet 4). Based on the PG‐SGA score, nutritional status is generally categorized into four groups^14^: well‐nourished (0–1 points), mildly malnourished (2–3 points), moderately malnourished (4–8 points), and severely malnourished (≥9 points).

### Item selection and generation of the Modified Patient‐Generated Subjective Global Assessment

We first conducted a survey on the use of the PG‐SGA among medical staff across the country. Seventy healthcare professionals (details shown in *Figure S1* and *Table S* *2*) from 23 Chinese provinces were asked to complete a questionnaire consisting of 24 questions (*Table S3*) on the content validity, comprehensibility and difficulty of completing the PG‐SGA (i.e. Boxes 1–4 and Worksheets 1–4). In addition, eight open‐ended questions were posed after each box and worksheet to ask for the professionals' perceptions regarding its utility.

The medical professionals were also asked about whether their patients or their patients' family members could complete the patient‐generated components (i.e. Boxes 1–4) independently during routine clinical work. After the results of the questionnaires were recorded, research meetings were set up to discuss which item(s) should be deleted or included or if new items should be added. The individual components (i.e., Boxes 1–4 and Worksheets 1–4) would be adjusted if more than 5% of professionals expressed doubts about their comprehensibility and would be deleted if more than 50% of professionals thought they were difficult to complete. After discussion, imperfect items were selected for further reliability analyses. Items were selected for inclusion if the item‐total correlation was >0.1 and Kendall's tau‐b rank correlation was >0.1; otherwise, the items were deleted. Finally, all selected items were used to generate the new mPG‐SGA.

### Validation in the Modified Patient‐Generated Subjective Global Assessment

The internal validity of the newly generated mPG‐SGA was first tested using Pearson's correlation analysis by comparing the total mPG‐SGA score and the scores from each section (i.e. Boxes 1–4, age). The external validity was examined using Pearson's correlation by comparing scores of the mPG‐SGA scores with the scored PG‐SGA, global PG‐SGA rating, NRS 2002, and KPS. For the test–retest validity, the mPG‐SGA was conducted in consented participants on a single day, followed by a repeated assessment by the same investigator and other professionals on a different day within 1 week. The assessment days were separated by at least 1 days without chemotherapy, surgery, or other operations that might obviously affect the results of the patient's nutritional assessment. The test–retest validity can be also examined by comparing scores of Box 4 of the mPG‐SGA with the KPS score and the ECOG PS score from the same patients.

The performance of the mPG‐SGA was compared with that of the PG‐SGA based on the area under the curve (AUC). The AUC of the abPG‐SGA (Boxes 1–4 of the PG‐SGA) was calculated. To compare the completion time of the questionnaire, the mPG‐SGA was first conducted in consented participants on a single day and then the PG‐SGA was conducted on the next day by the same professional. The ability of the mPG‐SGA to categorize patients with different nutritional statuses was also evaluated by comparing the average nutritional parameters of each group, such as the albumin, triceps skinfold thickness (for estimate of subcutaneous fat), and hand grip strength (for estimate of muscle strength).

Finally, the relationship between the nutritional status evaluated by the mPG‐SGA and patient overall survival (OS) was examined using Kaplan–Meier methods. OS was defined as the time from the date of diagnosis to the date of death from any cause, the date of the last follow‐up or 30 April 2019, whichever came first. The ability to predict survival based on the categorization of the patient's nutritional status was also simultaneously compared among mPG‐SGA, abPG‐SGA and the original PG‐SGA.

### Statistical analysis

The item‐total correlation and Cronbach's alpha coefficient were used to test the reliability and internal consistency of the PG‐SGA. Kendall's tau‐b was used to identify the rank correlation between each item and the variables in the PG‐SGA score (categorized into four ranges: 0–1, 2–3, 4–8, and ≥9). External and internal consistencies were examined by concordance analyses. The mPG‐SGA was validated using the original PG‐SGA. Receiver operating characteristic curves were used to compare the sensitivity and specificity of the ability to accurately identify different nutritional statuses across the mPG‐SGA box combinations. The sensitivity and specificity of the mPG‐SGA in comparison with the PG‐SGA were tested by AUC analyses. The cut‐off scores were calculated from the points of maximal specificity and sensitivity (Youden's index). When assessing the consistency between nutritional statuses determined by the mPG‐SGA, the abPG‐SGA and the original PG‐SGA, weighted Kappa values <0.5, 0.5–0.75, 0.75–0.9, and >0.9 were considered poor, moderate, good and excellent, respectively.^15^ Test–retest reliability was examined using Spearman correlation coefficients. Two related samples nonparametric test was used to compare scores and time. OS was evaluated by Kaplan–Meier methods. *P* values ≤0.05 (two‐sided) were considered statistically significant. All analyses were conducted using SPSS 26 (IBM Corp, Armonk, NY, USA).

### Role of the funding source

The funders of the study had no role in study design, data collection, data analysis, data interpretation, or writing of the report. The corresponding authors had full access to all the data in the study and had final responsibility for the decision to submit for publication.

## Results

The characteristics of patients recruited for this study are shown in *Table* 1. Overall, 82% (27 935 out of 34 071) of the participants were malnourished; 58% were moderately or severely malnourished. The average PG‐SGA scores and percentages of patients with moderate or severe malnutrition in different tumour locations are shown in *Table* *S1*. The percentages of patients with moderate or severe malnutrition ranged from 36% in breast cancer to 80.7% in oesophageal cancer.

**Table 1:** Patient characteristics

Characteristics	*n* (%)
Age, older than 65 years	8476 (24.9)
Sex, male	18 794 (55.2)
Primary tumour location	
Pancreatic cancer	468 (1.4)
Biliary cancer	121 (0.4)
Oesophageal cancer	2512 (7.4)
Gastric cancer	4517 (13.3)
GIST	45 (0.1)
Colorectal cancer	6686 (19.6)
Liver cancer	1315 (3.9)
Brain cancer	342 (1.0)
Leukaemia	871 (2.6)
Lung cancer	6913 (20.3)
Ovarian cancer	778 (2.3)
Malignant lymphoma	1009 (3.0)
Cervical cancer	1434 (4.2)
Endometrial cancer	404 (1.2)
Prostate cancer	298 (0.9)
Bladder cancer	270 (0.8)
Nasopharyngeal carcinoma	2308 (6.8)
Breast cancer	3687 (10.8)
Other cancer	1022 (3.0)
Nutritional status (PG‐SGA score)	
Well‐nourished (0–1 point)	6136 (18.0)
Mild malnutrition (2–3 points)	8084 (23.7)
Moderate malnutrition (4–8 points)	11 095 (32.6)
Severe malnutrition (≥9 points)	8756 (25.7)
Recent treatment	
Surgery	8756 (25.7)
Chemotherapy	17 399 (51.1)
Radiotherapy	4490 (13.2)
Use of nutritional support	25 017 (73.4)

PG‐SGA, Patient‐Generated Subjective Global Assessment.

**Figure 1:**
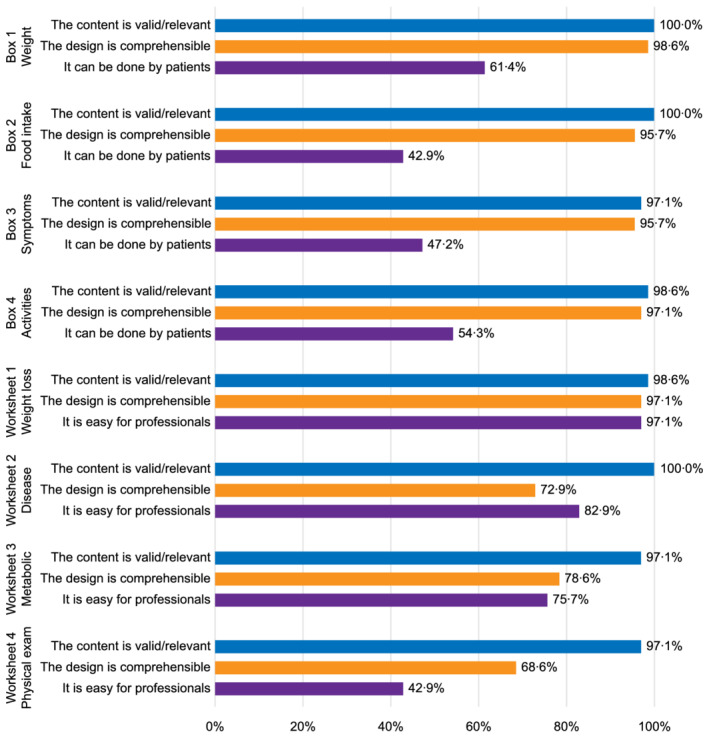
The content validity, comprehensibility, and difficulty of each component of the Patient‐Generated Subjective Global Assessment as perceived by healthcare professionals in China.

**Figure 2:**
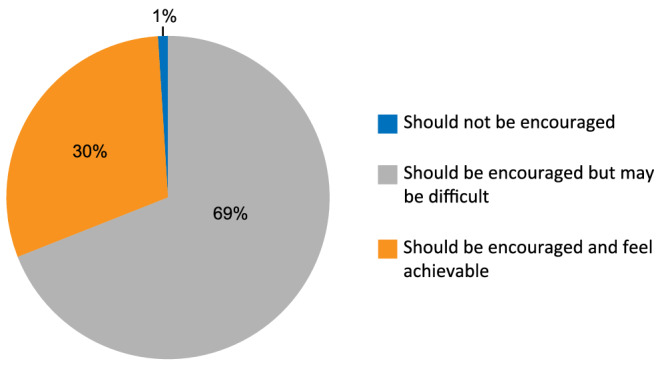
Results to the question ‘Should patients or their families be encouraged to complete the nutritional assessment by themselves?’.

*Figure* 1 shows the results of the evaluation of the original PG‐SGA questionnaire by healthcare professionals. In general, the assessment of Boxes 1–4 and Worksheet 1 of the PG‐SGA fell into the predefined acceptance category. Almost all professionals (>95%) acknowledged the content validity, comprehensibility, and low difficulty for professional completion of these parts of the assessment. However, the professionals often doubted that the patients and family members themselves would be able to complete the patient‐generated components without help from professionals (61%, 43%, 47%, and 54% for Boxes 1–4, respectively). Although 69% of professionals were concerned about it being difficult for patients or their families to complete the nutritional assessment by themselves, most professionals (99%) agreed that patients or their families should be encouraged to complete it by themselves (*Figure* 2); 69% to 79% of the professionals acknowledged the comprehensibility of Worksheets 2–4. However, 57% of professionals thought it was difficult for medical staff to complete Worksheet 4. Thus, the items in Worksheet 4 were deleted. More professional opinions are shown in *Table S4*.

As shown in *Table* 2, the internal consistency of PG‐SGA items was acceptable (Cronbach's alpha = 0.648). The components and items with an item‐total correlation <0.1 and Kendall's tau‐b rank correlation <0.1 was deleted (‘mouth sores’ and ‘other’ in Box 3, all items in Worksheet 2 except for ‘age greater than 65 years’, and items in Worksheet 3 of the PG‐SGA). Finally, the new mPG‐SGA (*Table* 3) was constructed by combining Boxes 1–4 and the item ‘age greater than 65 years’ and excluding items ‘mouth sores’ and ‘other’ in Box 3.

**Table 2:** Internal consistency of the PG‐SGA items^a^ and Kendall's tau‐b rank correlation between each item and categorized PG‐SGA level^b^

Items of PG‐SGA	Mean (SD)	Corrected item‐total correlation^a^	Cronbach's alpha if item deleted^a^	Kendall's tau‐b^b^
Box 1. Weight (& Worksheet 1)	1.11 (1.64)	0.407	0.627	0.597
Box 2. Food intake	0.73 (0.96)	0.593	0.574	0.656
Box 3. Symptoms				
No appetite	0.57 (1.18)	0.362	0.619	0.511
Nausea	0.10 (0.31)	0.345	0.634	0.312
Vomiting	0.18 (0.71)	0.289	0.629	0.294
Mouth sores	0.02 (0.22)	0.066	0.649	0.054
Constipation	0.08 (0.27)	0.216	0.642	0.212
Diarrhoea	0.11 (0.55)	0.103	0.649	0.191
Dry mouth	0.08 (0.27)	0.185	0.644	0.182
No taste	0.06 (0.23)	0.215	0.643	0.210
Smells bother me	0.02 (0.16)	0.155	0.647	0.141
Problems swallowing	0.11 (0.45)	0.157	0.644	0.223
Full quickly	0.06 (0.23)	0.195	0.644	0.218
Pain	0.29 (0.89)	0.224	0.640	0.336
Other	0.03 (0.17)	0.077	0.649	0.073
Box 4. Activities and function	0.47 (0.77)	0.471	0.603	0.473
Worksheet 2. Disease				
Age older than 65 years	0.25 (0.43)	0.108	0.647	0.143
Other diseases	0.04 (0.20)	0.012	0.651	0.025
Worksheet 3. Metabolic demand	0.06 (0.42)	0.090	0.649	0.069
Worksheet 4. Physical exam	0.54 (0.71)	0.374	0.618	0.307

PG‐SGA, Patient‐Generated Subjective Global Assessment.

^a^ By reliability statistics, Cronbach's alpha = 0.648.

^b^ Well‐nourished or mildly malnourished (0–3 points), moderately malnourished (4–8 points), and severely malnourished (≥9 points).

**Table 3:** Modified Patient‐Generated Subjective Global Assessment (mPG‐SGA)

Box 1. Weight	Wt loss past month	Points	Wt loss 6 months	Box 1. Score
1.1 A summary of my current and recent weight:	10% or greater	4	20% or greater	
I currently weigh about _______ pounds	5–9.9%	3	10–19.9%	
I am about ____ feet ____ inches tall	3–4.9%	2	6–9.9%	
One month ago, I weighed about _________ pounds	2–2.9%	1	2–5.9%	
Six months ago, I weighed about _________ pounds	0–1.9%	0	0–1.9%	
1.2 During the past two weeks my weight has:	Use the 1 month weight data if available. Use the 6 month data only if there is no 1 month weight data. Add one extra point if the patient has lost weight during the past 2 weeks.			
Decreased (1) not changed (0) increased (0)				

Box 2. Food Intake: Compared to my normal intake, I would rate my food intake during the past month as follows:	Box 2. Score
Unchanged (0)	Use the highest score checked, no matter how many are checked
More than usual (0)	
Less than usual (1)	
If less than usual, I am now eating:	
Normal food but less than the normal amount (1)	
Little solid food (2)	
Only liquids or nutritional supplements (3)	
Very little of anything (4)	
Only tube feedings or only nutrition by vein (0)	

Box 3. Symptoms: I have had the following problems that have kept me from eating enough during the past 2 weeks (check all that apply):	Box 3. Score
No problems eating (0)	Add all points for Box 3 to obtain a total score
No appetite, just did not feel like eating (3)	
I have had: nausea (1) vomiting (3) constipation (1) diarrhoea (3) dry mouth (1) problems swallowing (2) pain; where? (3) __________________	
Things taste funny or have no taste (1) Smells bother me (1)	
I feel full quickly (1)	

Box 4. Activities and function: Over the past month, I would generally rate my activity as:	Box 4. Score
Normal with no limitations (0)	
Not my normal self but able to be up and about with fairly normal activity (1)	
Not feeling up to most things but in bed or chair less than half the day (2)	
Able to do little activity and spend most of the day in bed or a chair (3)	
Pretty much bedridden, rarely out of bed (3)	

Box 5. Age		Box 5. Score
Age older than 65 years (1)		
0 = Well‐nourished; 1–2 = mildly malnourished; 3–6 = moderately malnourished; ≥7 = severely malnourished	Total score/level:	/

The internal consistency and external consistency were estimated by calculating the correlations of the mPG‐SGA score with the individual components and other performance scores (*Table* 4). The correlation between the mPG‐SGA score and the overall PG‐SGA rating (A, B, and C) was 0.625 (*P* < 0.001), and the correlation with the total PG‐SGA score was 0.984 (*P* < 0.001). The correlations between the mPG‐SGA score and its individual components were significant (*P* < 0.001) and were 0.684, 0.722, 0.842, 0.589, and 0.189 for Boxes 1–4 and ‘age more than 65’, respectively. The correlations with other performance scores (NRS 2002, KPS) were weaker but still significant (*P* < 0.001). Box 4 of the PG‐SGA (the activity and functioning of patients) asked the same questions as the KPS but in a different way. By re‐categorizing the KPS as an ECOG performance score (PS), the alternate‐form reliability was able to be examined. Good external consistency was shown between Box 4 of the PG‐SGA and the KPS and the ECOG PS (Pearson *r* = 0.626 and 0.568 respectively, *P* < 0.001) (*Table S5*). Test–retest reliability was investigated in 134 patients, which included 89 patients for inter‐rater and 45 for intra‐rater testing. No significant difference and strong correlations were observed in the total mPG‐SGA scores between two measurements by five independent raters (Spearman correlation coefficient = 0.964, *Table 6A*) and between the repeated measurements by the same professional (Spearman correlation coefficient = 0.995, *Table S6B*). The times needed to complete the questionnaire were recorded for 30 patients. The mean time to complete the mPG‐SGA was significantly shorter than the PG‐SGA [281 (±59) seconds vs. 411 (±77) seconds, *P* < 0.001] (*Table S7* ).

**Table 4:** Correlations (Pearson, *r*) of the total mPG‐SGA score with individual components and other indexes and performance scores

Total mPG‐SGA score	Total PG‐SGA score	Global PG‐SGA rating (A, B, C)	Box 1. weight loss	Box 2. food intake	Box 3. symptoms with 2 deleted	Box 4. activities and function	Age older than 65	NRS 2002 score	KPS
Pearson *r*	0.984	0.625	0.684	0.722	0.842	0.589	0.189	0.501	0.441
*P*	<0.001	<0.001	<0.001	<0.001	<0.001	<0.001	<0.001	<0.001	<0.001
*N*	34 071	34 071	34 071	34 071	34 071	34 071	34 071	34 071	34 071

Abbreviations: KPS, Karnofsky performance status; PG‐SGA, Patient‐Generated Subjective Global Assessment; mPG‐SGA, modified PG‐SGA.

The combinations of box scores that met the sensitivity and specificity criteria are described in *Table* 5. Different cut‐off scores were used for these variables to determine the best sensitivity, specificity, and accuracy compared with the categorized PG‐SGA scores. Four nutritional status classifications were formed, that is, well‐nourished (0 points), mildly malnourished (1–2 points), moderately malnourished (3–6 points), and severely malnourished (≥7 points), based on the analyses of the area under curve (0.962, 0.989, and 0.985 for the three cut‐off scores, respectively) and the point of maximal sensitivity (0.924, 0.918, and 0.945) and specificity (1.000, 1.000, and 0.938) of the mPG‐SGA scores. Compared with the original PG‐SGA, the mPG‐SGA showed better consistency than the abPG‐SGA (weighted kappa: 0.881 vs. 0.830). However, the accuracy was reduced when the weight loss score (Box 1) was removed (weighted kappa <0.8). We evaluated the diagnostic consistency between mPG‐SGA and PG‐SGA for 18 types of malignant tumours. The weighted kappas were ranged from 0.854 to 0.946, which indicated excellent consistency (*Table S8*). Thus, the items selected for inclusion in the mPG‐SGA and the scoring were suitable in most common tumour types. The ability of the mPG‐SGA to categorize patients into different nutritional status groups according to the anthropometric parameters is shown in *Table S9*. Again, the discriminatory ability of the majority of the nutritional parameters was highly significant based on the evaluation of these objective parameters (*P* < 0.001).

**Table 5:** Area under receiver operating characteristic curve and the sensitivity, specificity and agreement with the PG‐SGA in the prediction of malnutrition (PG‐SGA well‐nourished or mildly, moderately, or severely malnourished)

Method		Well‐nourished or mildly malnourished (PG‐SGA score 0–1 points or more)^a^				Mildly or moderately malnourished (PG‐SGA score 0–3 points or more)^a^				Moderately or severely malnourished (PG‐SGA score 4–8 points or more)^a^				Weighted
		AUC	Cut‐off score	Sensitivity	Specificity	AUC	Cut‐off score	Sensitivity	Specificity	AUC	Cut‐off score	Sensitivity	Specificity	kappa
A.	abPG‐SGA (Boxes 1–4)^b^	0.927	0.5	0.854	1.000	0.986	1.5	0.965	0.935	0.980	6.5	0.905	0.944	0.830
B.	mPG‐SGA	0.962	0.5	0.924	1.000	0.989	2.5	0.918	1.000	0.985	6.5	0.945	0.938	0.881
C.	mPG‐SGA‐Box 1	0.933	0.5	0.866	1.000	0.921	1.5	0.809	0.939	0.887	4.5	0.787	0.819	0.739
D.	mPG‐SGA‐Box 1 + Worksheet 4	0.975	0.5	0.951	1.000	0.935	1.5	0.862	0.899	0.905	4.5	0.855	0.774	0.777

Abbreviations: abPG‐SGA, abridged PG‐SGA; AUC, area under the curve; CI, confidence interval; mPG‐SGA, Modified PG‐SGA; PG‐SGA, Patient‐Generated Subjective Global Assessment; Worksheet 4: physical exam; Box 1: weight loss.

^a^ The overall agreement with the PG‐SGA was calculated by the receiver operating characteristic area under the curve.

^b^ Boxes 1–4 included components of weight loss, food intake, symptoms, and activities and functions.

**Figure 3:**
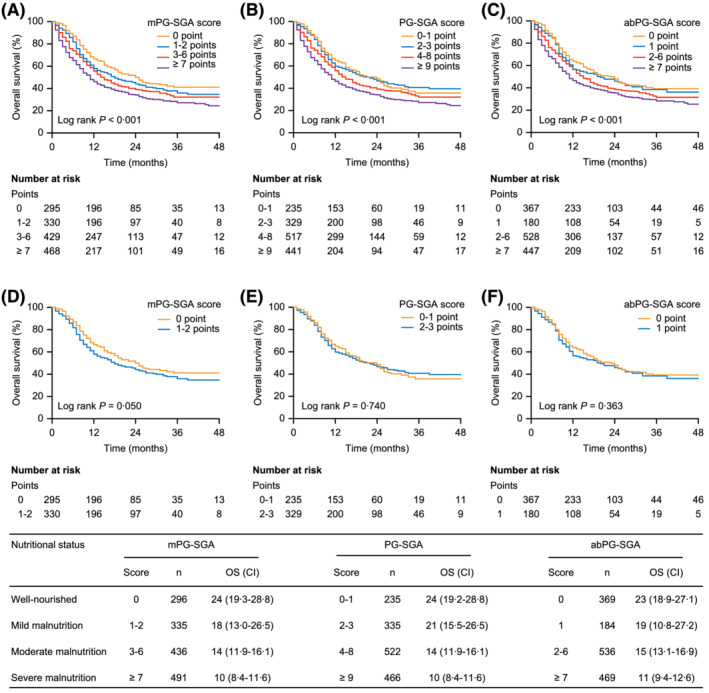
Kaplan–Meier curves for the OS of patients in different nutritional status groups diagnosed by the mPG‐SGA (*A*,*D*), PG‐SGA (*B*,*E*), or abPG‐SGA (*C*,*F*). The OS rates between different nutritional status groups were analysed and compared by Kaplan–Meier analysis and log‐rank test. Abbreviations: abPG‐SGA, abridged PG‐SGA; CI, confidence interval; OS, overall survival; mPG‐SGA, modified PG‐SGA; PG‐SGA, Patient‐Generated Subjective Global Assessment.

In this study, 1558 patients were followed up for five years, and 1,042 died during this period. The Kaplan–Meier curve analysis shows that the survival rates were significantly different among the groups of patients with different nutritional statuses categorized by the mPG‐SGA (*Figure* 3). The median OS time was 24, 18, 14, and 10 months for patients who were well‐nourished (0 points), mildly malnourished (1–2 points), moderately malnourished (3–6 points), and severely malnourished (≥7 points), respectively (*P* < 0.001). It should be noted that no survival difference was shown between patients diagnosed as being well nourished and those diagnosed as being mildly malnourished by the original PG‐SGA (*P* = 0.740) and abPG‐SGA (*P* = 0.363), but the difference could be detected by the mPG‐SGA (*P* = 0.050). As shown in *Figure* 3A–3C, the survival curves for well‐nourished and mildly malnourished patients were clearly separated by the mPG‐SGA but not by the PG‐SGA or the abPG‐SGA.

## Discussion

In this study, we developed and validated the mPG‐SGA, a modified version of the PG‐SGA that was customized for assessing nutritional status of cancer patients. To our knowledge, this is the first time a nutritional assessment tool has been designed and validated using the data from the INSCOC, the largest retrospective nutrition cohort of cancer patients. In addition, the simplicity and the better predictive validity of the mPG‐SGA indicate that it may be an ideal nutritional assessment tool for cancer patients.

Recently, increasing emphasis has been placed on the nutritional status of cancer patients.^16^ The INSCOC study explored the malnutrition prevalence in cancer patients in China and its relationship to quality of life and other clinical outcomes.^13^ In the INSCOC, the PG‐SGA was used to assess the patients' nutritional status. The original PG‐SGA has been applied in China for more than 17 years.^17^ The PG‐SGA generally works well for assessing the nutritional status of cancer patients.^18^, ^19^ However, there have been a variety of issues in its application noted by both professionals and patients. The most pressing challenge is that it is difficult to implement routine 10 min nutritional assessment using the original PG‐SGA in busy, resource‐stretched units. The abPG‐SGA and other short‐form tools were built to address this issue.^2^, ^7^, ^8^ However, the abPG‐SGA was generated by simply deleting the professional‐generated section of the PG‐SGA without rigorous examination of the effects of this deletion.^7^, ^20^ In addition, these tools were designed for screening for malnutrition, not for nutritional assessment.^7^, ^10^, ^20^ Thus, we systematically develop this simpler version of the PG‐SGA as a nutritional assessment tool for cancer patients.

When the medical professionals were asked for feedback, all of them acknowledged that the tool helped to increase nutritional awareness^21^ as a result of encouraging patients to complete the self‐assessment items of the patient‐generated section. However, fewer than half of the professionals thought it was feasible for patients and family members to complete the self‐assessment independently. Wrong answers often occurred when patients and family members failed to understand the questions correctly. For example, some participants failed to read the whole sentence; and some selected more than one response option for questions which they were supposed to select only one simply because they felt that only one option did not fully capture their true situation.^11^ Therefore, although patient‐generated components can be completed by patients or their family members, we still proposed involvement of medical staff in the completion of the mPG‐SGA.

The selection of the items for inclusion in the mPG‐SGA was also based on rigorous statistical analyses. The comprehensibility and difficulty of the patient‐generated section were considered to be excellent, while the professional component was perceived to be below acceptable, with the physical exam being rated the most difficult component.^9^, ^22^ Worksheet 4 (physical exam) was removed from the mPG‐SGA because >50% of professionals thought it was difficult to obtain this information. This finding is similar to what has been found in previous studies.^9^ We tried to add BMI as an item in the mPG‐SGA to compensate for the lack of items from the physical examination. However, the diagnostic consistency of the mPG‐SGA with the PG‐SGA was not increased by this addition, and the specificity was decreased. In fact, patients receiving chemotherapy or anti‐angiogenesis treatment may experience significant weight gain due to ascites and oedema, making the body weight a poor indicator of the nutritional status.^23^ Therefore, we did not include BMI in the mPG‐SGA. Although some professionals stated that the exact weight loss of patients in 1 or 6 months cannot always be obtained, an unquantified weight loss without a timeframe is considered by many other nutritional screening or assessment tools as a better option than removing this important item.^6^ Hence, we kept the item of weight loss and allowed for the use of an estimated weight if the accurate value could not be obtained in Box 1. Food intake (Box 2) and assessment of function (Box 4) were easy to answer and were included by most nutritional screening or assessment tools.^6^ In this study, Box 3 (symptoms) of the PG‐SGA contributed most to the explained variance in nutritional status, which is also similar to findings from other countries.^24^, ^25^ Similar to the validation in Thai setting, the consistency between the mPG‐SGA and full PG‐SGA decreased when we deleted any of the boxes (1–4) from the mPG‐SGA.^3^ We thus kept all the boxes (1–4) in the mPG‐SGA.

The internal validity and the external validity between each part of the mPG‐SGA compared with the PG‐SGA, NRS 2002, and KPS were good. The mPG‐SGA fitted well with the original PG‐SGA with a better consistency than all the other nutritional tools.^6^ The good test–retest reliability of mPG‐SGA reflects the sound stability of the questionnaire. By re‐categorizing the KPS into an ECOG PS, we also evaluated the alternate‐form reliability. As the similar questions were asked several minutes apart, test–retest reliability was also virtually evaluated. Using mPG‐SGA, the mean nutritional assessment time was 281 s, which was significantly shorter than 411 s using PG‐SGA (*P* < 0.001). The 5 year survival rate in this study was low, about 30%. This is due to the lower 5 year survival rate in Chinese cancer patients (about 40.5% in 2015).^26^ Because this study only recruited patients from tertiary hospitals, there were patients with more severe conditions than that of the overall cancer population; this might be also responsible for the lower survival rate. In this study, and many others, neither the PG‐SGA nor the abPG‐SGA detected survival differences between patients categorized as well‐nourished and those categorized as mildly malnourished.^5^, ^27^ However, a significant survival difference was found between these two nutritional status groups when the mPG‐SGA was used for categorization. Therefore, the mPG‐SGA and its current cut‐offs might be optimal and enable better prediction of patient survival, and might replace the PG‐SGA for the assessment of malnutrition among cancer patients because better discrimination of OS is the ultimate goal for developing a new tool.

The current study has the strength of the largest sample size. To date, most PG‐SGA validation studies were conducted on small samples.^9^ Although the PG‐SGA was used to categorize patients into different nutritional status groups, the criteria used for categorizing the nutritional status differed among studies.^28^, ^29^ Our sample size was large enough to allow for the inclusion of a sufficient number of patients with different cancer types; additionally, we were able to obtain a stable estimate and determine the best cut‐offs for mPG‐SGA scores to categorize nutritional status. The items included in the mPG‐SGA are simple items that can be answered by patients. Although it is not currently recommended that patients be allowed to answer questions independently, we hope that future studies reveal the simplicity and accuracy of the mPG‐SGA and make it possible to achieve this goal. When combined with clinical data, the mPG‐SGA can easily inform the physician of a patient's nutritional status and thus permit suitable and timely intervention.^30^

One limitation of the present study is that there is no gold standard to estimate a patient's nutritional status. Thus, the criterion validity of mPG‐SGA could not be evaluated. As this was mainly a cross‐sectional study, the probabilities of confounding factors and bias were unavoidable. Although we included patients with a wide variety of cancers and recruited health‐care professionals and cancer patients from all over China, the findings might be representative of the situation of tertiary hospitals in China at most. The results are not necessarily generalizable to other populations. Furthermore, the present observations need to be examined in a longer follow‐up. Similar studies in other populations and countries are warranted. Extensive international cooperation and collaboration are needed to further refine and validate the mPG‐SGA.

## Conclusions

We systematically developed and validated a new modified version of the PG‐SGA, the mPG‐SGA. It appears to be a valid tool to assess the nutritional status of cancer patients. The mPG‐SGA may also provide better prognostic information for OS than both the full PG‐SGA and abPG‐SGA. This project has the potential to profoundly impact nutritional oncology worldwide following validation and refinement in future international research.

## Conflict of interest

The authors declare no conflicts of interest.

## Funding

The National Key Research and Development Program: The key technology of palliative care and nursing for cancer patients (2017YFC1309200).

The National Science Foundation of China (81773555).

## Supporting information


**Table S1.** Average PG‐SGA scores and percentages of patients with moderate or severe malnutrition by tumour location.
**Table S2.** Characteristics of the healthcare professionals who completed the questionnaires.
**Table S3.** Questionnaire survey of medical staff members about the PG‐SGA.
**Table S4.** Expert opinions regarding the selection of items.
**Table S5.** Correlations of the Box 4 score (Pearson, r) with the KPS and PS.
**Table S6a.** Inter‐rater reliability test of the mPG‐SGA in 89 patients.
**Table S6b.** Intra‐rater reliability test of the mPG‐SGA in 45 patients.
**Table S7.** The comparison of the completion time (seconds) of the mPG‐SGA and the PG‐SGA.
**Table S8.** Area under the receiver operating characteristic curve, and the sensitivity, specificity and agreement between the mPG‐SGA and the PG‐SGA (well‐nourished or mildly, moderately, and severely malnourished)*.
**Table S9.** Characteristics of the patients based on the nutritional assessment according to the mPG‐SGA (*N* = 34,071).
**Figure S1.** The locations where the professionals worked.

## References

[1] Mendes NP , Barros TA , Rosa COB , Franceschini S . Nutritional screening tools used and validated for cancer patients: a systematic review. Nutr Cancer 2019;71:898–907. 31033348 10.1080/01635581.2019.1595045

[2] Mauricio SF , Xiao J , Prado CM , Gonzalez MC , Correia M . Different nutritional assessment tools as predictors of postoperative complications in patients undergoing colorectal cancer resection. Clin Nutr 2018;37:1505–1511. 28918167 10.1016/j.clnu.2017.08.026

[3] Nitichai N , Angkatavanich J , Somlaw N , Voravud N , Lertbutsayanukul C . Validation of the scored patient‐generated subjective global assessment (PG‐SGA) in Thai setting and association with nutritional parameters in cancer patients. Asian Pac J Cancer Prev 2019;20:1249–1255. 31030501 10.31557/APJCP.2019.20.4.1249 PMC6948895

[4] Wiegert EVM , Padilha PC , Peres WAF . Performance of patient‐generated subjective global assessment (PG‐SGA) in patients with advanced cancer in palliative care. Nutr Clin Pract 2017;32:675–681. 28850795 10.1177/0884533617725071

[5] Martin L , Watanabe S , Fainsinger R , Lau F , Ghosh S , Quan H , et al. Prognostic factors in patients with advanced cancer: use of the patient‐generated subjective global assessment in survival prediction. J Clin Oncol 2010;28:4376–4383. 20805456 10.1200/JCO.2009.27.1916

[6] Miller J , Wells L , Nwulu U , Currow D , Johnson MJ , Skipworth RJE . Validated screening tools for the assessment of cachexia, sarcopenia, and malnutrition: a systematic review. Am J Clin Nutr 2018;108:1196–1208. 30541096 10.1093/ajcn/nqy244

[7] Vigano AL , di Tomasso J , Kilgour RD , Trutschnigg B , Lucar E , Morais JA , et al. The abridged patient‐generated subjective global assessment is a useful tool for early detection and characterization of cancer cachexia. J Acad Nutr Diet 2014;114:1088–1098. 24462323 10.1016/j.jand.2013.09.027

[8] Kim JY , Wie GA , Cho YA , Kim SY , Kim SM , Son KH , et al. Development and validation of a nutrition screening tool for hospitalized cancer patients. Clin Nutr 2011;30:724–729. 21813215 10.1016/j.clnu.2011.06.001

[9] Erickson N , Storck LJ , Kolm A , Norman K , Fey T , Schiffler V , et al. Tri‐country translation, cultural adaptation, and validity confirmation of the scored patient‐generated subjective global assessment. Support Care Cancer 2019;27:3499–3507. 30684046 10.1007/s00520-019-4637-3

[10] Gabrielson DK , Scaffidi D , Leung E , Stoyanoff L , Robinson J , Nisenbaum R , et al. Use of an abridged scored patient‐generated subjective global assessment (abPG‐SGA) as a nutritional screening tool for cancer patients in an outpatient setting. Nutr Cancer 2013;65:234–239. 23441610 10.1080/01635581.2013.755554

[11] Balstad TR , Bye A , Jenssen CR , Solheim TS , Thoresen L , Sand K . Patient interpretation of the patient‐generated subjective global assessment (PG‐SGA) short form. Patient Prefer Adherence 2019;13:1391–1400. 31496666 10.2147/PPA.S204188 PMC6701615

[12] Abbott J , Teleni L , McKavanagh D , Watson J , McCarthy AL , Isenring E . Patient‐generated subjective global assessment short form (PG‐SGA SF) is a valid screening tool in chemotherapy outpatients. Support Care Cancer 2016;24:3883–3887. 27095352 10.1007/s00520-016-3196-0

[13] Xu H , Song C , Wang C , Fu Z , Guo Z , Lin Y . Investigation on nutrition status and clinical outcome of patients with common cancers in Chinese patients: a multicenter prospective study protocol. Int J Clin Trials 2020;2:1–9.

[14] Ottery FD . Definition of standardized nutritional assessment and interventional pathways in oncology. Nutrition 1996;12:S15–S19. 8850213 10.1016/0899-9007(96)90011-8

[15] Koo TK , Li MY . A guideline of selecting and reporting intraclass correlation coefficients for reliability research. J Chiropr Med 2016;15:155–163. 27330520 10.1016/j.jcm.2016.02.012 PMC4913118

[16] Muscaritoli M , Arends J , Aapro M . From guidelines to clinical practice: a roadmap for oncologists for nutrition therapy for cancer patients. Ther Adv Med Oncol 2019;11:1758835919880084. 31762796 10.1177/1758835919880084 PMC6854759

[17] Hanping S , Wei L , Yumei Q , Weixin C . Nutrition screening and assessment. Beijing, China: The Peoples Medical Publishing House; 2014.

[18] Zhenming F , Hongxia X , Chunhua S , Wei L , Zengqing G , Yuan L , et al. Validity of the Chinese version of the patient‐generated subjective global assessment (PG‐SGA) in lung cancer patients. J Nutr Oncol 2016; 1(1): 52–59. http://www.nutroncol.com/front/article.html?ar=a_20180419163330402

[19] Zhenming F , Hongxia X , Chunhua S , Wei L , Zengqin G , Yuan L , et al. Validity of the Chinese version of the patient‐generated subjective global assessment (PG‐SGA) in gastric cancer patients. J Nutr Oncol 2018; 3(4): 182–188.

[20] Shahvazi S , Onvani S , Heydari M , Mehrzad V , Nadjarzadeh A , Fallahzadeh H . Assessment of nutritional status using abridged scored patient‐generated subjective global assessment in cancer patient. J Cancer Res Ther 2017;13:514–518. 28862219 10.4103/0973-1482.177500

[21] Jager‐Wittenaar H , de Bats HF , Welink‐Lamberts BJ , Gort‐van Dijk D , van der Laan BF , Ottery FD , et al. Self‐completion of the patient‐generated subjective global assessment short form is feasible and is associated with increased awareness on malnutrition risk in patients with head and neck cancer. Nutr Clin Pract 2019;35:353–362. 31134665 10.1002/ncp.10313 PMC7078954

[22] Sealy MJ , Hass U , Ottery FD , van der Schans CP , Roodenburg JLN , Jager‐Wittenaar H . Translation and cultural adaptation of the scored patient‐generated subjective global assessment: an interdisciplinary nutritional instrument appropriate for Dutch cancer patients. Cancer Nurs 2018;41:450–462. 28538001 10.1097/NCC.0000000000000505

[23] Um MH , Choi MY , Lee SM , Lee IJ , Lee CG , Park YK . Intensive nutritional counseling improves PG‐SGA scores and nutritional symptoms during and after radiotherapy in Korean cancer patients. Support Care Cancer 2014;22:2997–3005. 24906838 10.1007/s00520-014-2304-2

[24] Kosters CM , van den Berg MGA , van Hamersvelt HW . Sensitive and practical screening instrument for malnutrition in patients with chronic kidney disease. Nutrition 2020;72:110643. 31926378 10.1016/j.nut.2019.110643

[25] Segura A , Pardo J , Jara C , Zugazabeitia L , Carulla J , de Las Peñas R , et al. An epidemiological evaluation of the prevalence of malnutrition in Spanish patients with locally advanced or metastatic cancer. Clin Nutr 2005;24:801–814. 15993517 10.1016/j.clnu.2005.05.001

[26] Zeng H , Chen W , Zheng R , Zhang S , Ji JS , Zou X , et al. Changing cancer survival in China during 2003–15: a pooled analysis of 17 population‐based cancer registries. Lancet Glob Health 2018;6:e555–e567. 29653628 10.1016/S2214-109X(18)30127-X

[27] Gallois C , Artru P , Lièvre A , Auclin E , Lecomte T , Locher C , et al. Evaluation of two nutritional scores' association with systemic treatment toxicity and survival in metastatic colorectal cancer: an AGEO prospective multicentre study. Eur J Cancer 2019;119:35–43. 31415985 10.1016/j.ejca.2019.07.011

[28] Li R , Wu J , Ma M , Pei J , Song Y , Zhang X , et al. Comparison of PG‐SGA, SGA and body‐composition measurement in detecting malnutrition among newly diagnosed lung cancer patients in stage IIIB/IV and benign conditions. Med Oncol 2011;28:689–696. 20422319 10.1007/s12032-010-9534-z

[29] Bahl A , Elangovan A , Kaur S , Verman R , Oinam AS , Ghoshal S , et al. Pre‐treatment nutritional status and radiotherapy outcome in patients with locally advanced head and neck cancers. Gulf J Oncolog 2017;1:61–63. 29019332

[30] de Bruin JS , Schuh C , Seeling W , Luger E , Gall M , Hütterer E , et al. Assessing the feasibility of a mobile health‐supported clinical decision support system for nutritional triage in oncology outpatients using Arden syntax. Artif Intell Med 2018;92:34–42. 26563776 10.1016/j.artmed.2015.10.001

[31] von Haehling S , Morley JE , Coats AJS , Anker SD . Ethical guidelines for publishing in the *Journal of Cachexia, Sarcopenia and Muscle*: update 2019. J Cachexia Sarcopenia Muscle 2019;10:1143–1145. 31661195 10.1002/jcsm.12501 PMC6818444

