# Supplementary material for: Development and validation of a Modified Patient‐Generated Subjective Global Assessment as a nutritional assessment tool in cancer patients
Source: J Cachexia Sarcopenia Muscle. 2021 Dec 4;13(1):343–54. doi: 10.1002/jcsm.12872 (PMC8818590; doi:10.1002/jcsm.12872)
Supplement: Supplementary file 1 — Table S1. Average PG‐SGA scores and percentages of patients with moderate or severe malnutrition by tumour location. Table S2. Characteristics of the healthcare professionals who completed the questionnaires. Table S3. Questionnaire survey of medical staff members about the PG‐SGA. Table S4. Expert opinions regarding the selection of items. Table S5. Correlations of the Box 4 score (Pearson, r) with the KPS and PS. Table S6a. Inter‐rater reliability test of the mPG‐SGA in 89 patients. Table S6b. Intra‐rater reliability test of the mPG‐SGA in 45 patients. Table S7. The comparison of the completion time (seconds) of the mPG‐SGA and the PG‐SGA. Table S8. Area under the receiver operating characteristic curve, and the sensitivity, specificity and agreement between the mPG‐SGA and the PG‐SGA (well‐nourished or mildly, moderately, and severely malnourished)*. Table S9. Characteristics of the patients based on the nutritional assessment according to the mPG‐SGA (N = 34,071). Figure S1. The locations where the professionals worked. [file JCSM-13-343-s001.docx]

**Supplemental Table 1.** Average PG-SGA scores and percentages of patients with moderate or severe malnutrition by tumour location

| **Primary tumour location** | **n** | **Mean (95% CI)** | **Percentage with moderate or severe malnutrition (%)** |
| --- | --- | --- | --- |
| Pancreatic cancer | 468 | 10.6 (10.1-11.2) | 80.60 |
| Biliary cancer | 121 | 9.5 (8.6-10.4) | 80.20 |
| Oesophageal cancer | 2512 | 8.8 (8.6-9.0) | 80.70 |
| Gastric cancer | 4517 | 8.8 (8.7-9.0) | 77.90 |
| GIST | 45 | 7.1 (5.7-8.5) | 66.70 |
| Colorectal cancer | 6686 | 6.9 (6.8-7.0) | 61.70 |
| Liver cancer | 1315 | 7.4 (7.1-7.7) | 60.90 |
| Brain cancer | 342 | 6.8 (6.3-7.4) | 58.80 |
| Leukaemia | 871 | 6.5 (6.2-6.8) | 58.70 |
| Lung cancer | 6913 | 6.5 (6.4-6.6) | 56.30 |
| Ovarian cancer | 778 | 6.6 (6.3-6.9) | 56.00 |
| Malignant lymphoma | 1009 | 6.3 (6.0-6.6) | 54.40 |
| Cervical cancer | 1434 | 6.0 (5.8-6.3) | 51.50 |
| Endometrial cancer | 404 | 5.4 (5.0-5.8) | 47.30 |
| Prostate cancer | 298 | 5.9 (5.4-6.4) | 43.00 |
| Bladder cancer | 270 | 5.7 (5.1-6.2) | 41.50 |
| Nasopharyngeal carcinoma | 2308 | 5.1 (4.9-5.3) | 36.40 |
| Breast cancer | 3687 | 4.6 (4.4-4.7) | 35.70 |
| Other cancer | 1022 | 6.6 (6.3-6.8) | 57.50 |

Abbreviations: PG-SGA, Patient-Generated Subjective Global Assessment; CI, confidence interval

**Supplemental Table 2.** Characteristics of the healthcare professionals who completed the questionnaires

|  | | **n** | **%** |
| --- | --- | --- | --- |
| Age group | |  |  |
|  | 20-29 | 2 | 2.86 |
|  | 30-39 | 12 | 17.14 |
|  | 40-49 | 29 | 41.43 |
|  | 50-59 | 25 | 35.71 |
|  | 60-69 | 2 | 2.86 |
| Occupation | |  |  |
|  | Nurse | 1 | 1.43 |
|  | Doctor | 40 | 57.14 |
|  | Nutritionist | 25 | 35.71 |
|  | Nutrition-related worker | 2 | 2.86 |
|  | Other | 2 | 2.86 |
| Title | |  |  |
|  | No title | 1 | 1.43 |
|  | Junior title | 2 | 2.86 |
|  | Intermediate title | 15 | 21.43 |
|  | Senior title | 52 | 74.29 |

**Supplemental Table 3.** Questionnaire survey of medical staff members about the PG-SGA

| Your age: |
| --- |
| Your occupation: |
| Your title: |
|  |
| Is the content of Box 1 valid? |
| Is Box 1 comprehensible? |
| Do you think that Box 1 can be filled in by the patient or family members themselves? |
| Open question: opinion about Box 1. |
| Is the content of Box 2 valid? |
| Is Box 2 comprehensible? |
| Do you think that Box 2 can be filled in by the patient or family members themselves? |
| Open question: opinion about Box 2 |
| Is the content of Box 3 valid? |
| Is Box 3 comprehensible? |
| Do you think that Box 3 can be filled in by the patient or family members themselves? |
| Open question: opinion about Box 3 |
| Is the content of Box 4 valid? |
| Is Box 4 comprehensible? |
| Do you think that Box 4 can be filled in by the patient or family members themselves? |
| Open question: opinion about Box 4. |
| Do you think that, in general, in the current hospital environment in China, should patients and their families be encouraged to complete Boxes 1-4 by themselves? |
|  |
| Is the content of Worksheet 1 valid? |
| Is Worksheet 1 comprehensible? |
| Do you think it is easy to complete Worksheet 1? |
| Open question: opinion about Worksheet 1. |
| Is the content of Worksheet 2 valid? |
| Is Worksheet 2 comprehensible? |
| Do you think it is easy to complete Worksheet 2? |
| Open question: opinion about Worksheet 2. |
| Is the content of Worksheet 3 valid? |
| Is Worksheet 3 comprehensible? |
| Do you think it is easy to complete Worksheet 3? |
| Open question: opinion about Worksheet 3. |
| Is the content of Worksheet 4 valid? |
| Is Worksheet 4 comprehensible? |
| Do you think it is easy to complete Worksheet 4? |
| Open question: opinion about Worksheet 4. |

**Supplemental Table 4.** Expert opinions regarding the selection of items

| **Opinion** | **Solution** |
| --- | --- |
| Box 1. Weight and Worksheet 1. Score for weight loss | |
| Patients may not notice a change from their previous weight. | The kappa value decreased from 0.909 (Cut-off, 2.5; 6.5) to 0.722 (Cut-off, 1.5; 4.5) when removed. It is better to keep this item. |
| Box 2. Food intake |  |
| The “3 points for oral nutritional supplements and 0 points for intravenous infusion supplements” are not suitable in China. Clinical nutritional support may not meet human needs. | Since the scores of ③④ are the same, they can be merged. It is difficult to reflect whether only tube feeding or nutrition by vein is sufficient in the questionnaire, and the patients who choose this item have a higher total PG-SGA score, so we will not change the scores, but recommend focusing on nutritional education for the family of the patients who selected this option. |
| Combine ③④ into liquid foods because patients and most medical staff cannot figure out what ‘liquids’ refers to. |  |
| Box 3. Symptoms |  |
| 1. Medically implemented fasting was not described; 2. It is recommended that the other options have a wider coverage | We carefully analysed the contents filled in the "other" option and found that it was mainly medical fasting or symptoms unrelated to the current disease. Additionally, since the total PG-SGA score was always ≥ 9 after medical fasting, the higher score did not change the patient's nutritional level, so we did not add more related symptoms. The item-total correlation for “mouth sores” and “other” were less than 0.1, so they were removed. |
| Some symptoms are not necessarily the symptoms caused by the disease. They may have already been present before the disease. |  |
| Worksheet 2. Disease |  |
| Due to the low awareness of one’s disease condition in China, the score may be inaccurate. | We deleted all items in worksheet 2 except for “age older than 65 years” because the item-total correlation and Kendall's tau-b rank correlation of these were less than 0.1. |
| Worksheet 3. Metabolic demand |  |
| Too complicated. | We chose to delete this part because their item-total correlation and Kendall's tau-b rank correlation were less than 0.1. |
| Very few patients have scores in this section, and the items are recommended for removal. |  |
| Worksheet 4. Physical exam |  |
| Assessing the degree of consumption is difficult, medical staff need to be trained, and assessments vary widely among different staff. | We chose to delete this section because more than 50% of professionals thought it was difficult to complete. |
| Total level assessment |  |
| The nutrition support classification recommendations are not very clear. | We prefer to use a 3-level assessment based on the overall PG-SGA scores: well-nourished or mildly malnourished (1-3 points), moderately malnourished (4-8 points), and severely malnourished (≥ 9 points). |
| “Is it possible to divide into 3 sections from 0-3, 4-8, 9 or more points?” |  |

**Supplemental Table 5.** Correlations of the Box 4 score (Pearson, r) with the KPS and PS

|  |  | **KPS** | **PS** |
| --- | --- | --- | --- |
| **Box 4** | N | 34071 | 34071 |
|  | Pearson, *r* | 0.626 | 0.568 |
|  | *P* | < 0.001 | < 0.001 |

Abbreviations: KPS, Karnofsky; PS, Eastern Cooperative Oncology Group performance status.

| **Supplemental Table 6a. Inter-rater reliability test of the mPG-SGA in 89 patients.** | | | | | | |
| --- | --- | --- | --- | --- | --- | --- |
| **Items** | **Total score** | **Box 1** | **Box 2** | **Box 3** | **Box 4** | **Box 5** |
| **mPG-SGA 1** | 4 (1, 9.5) | 0 (0, 3) | 0 (0, 1) | 1 (0, 4) | 1 (0, 1) | 0 (0, 1) |
| **mPG-SGA 2** | 4 (1, 9) | 0 (0, 3) | 0 (0, 1) | 1 (0, 3) | 1 (0, 2) | 0 (0, 1) |
| ***P**** | 0.058 | 0.477 | 0.204 | 0.007 | 0.011 | 1.000 |
| **Spearman correlation†** | 0.964** | 0.957** | 0.879** | 0.908** | 0.944** | 1.000** |
| Two measurements of the mPG-SGA (mPG-SGA 1 and mPG-SGA 2) were conducted by five independent raters on different days within one week. The details on the scores for each box are presented as median (25th percentile, 75th percentile). | | | | | | |
| *Two related samples nonparametric test was used to compare scores. | | | | | | |
| †Test-retest reliability was examined using Spearman correlation coefficients. | | | | | |  |

| **Supplemental Table 6b. Intra-rater reliability test of the mPG-SGA in 45 patients.** | | | | | | |
| --- | --- | --- | --- | --- | --- | --- |
| **Items** | **Total score** | **Box 1** | **Box 2** | **Box 3** | **Box 4** | **Box5** |
| **mPG-SGA day 1** | 8 (5, 9) | 0.5 (0, 3) | 1 (0, 2) | 3 (1, 4) | 1.5 (0, 3) | 0 (0, 1) |
| **mPG-SGA day 2** | 8 (5, 9) | 0.5 (0, 3) | 1 (0, 2) | 3 (1, 4) | 1.5 (0, 3) | 0 (0, 1) |
| ***P**** | 0.564 | 1.000 | 1.000 | 1.000 | 0.317 | 1.000 |
| **Spearman correlation†** | 0.995** | 1.000** | 1.000** | 0.988** | 0.985** | 1.000** |
| The mPG-SGA were conducted by one professional on different days (day 1 and day 2) within one week. The details on the scores for each box are presented as median (25th percentile, 75th percentile). | | | | | | |
| *Two related samples nonparametric test was used to compare scores. | | | | | | |
| †Test-retest reliability was examined using Spearman correlation coefficients. | | | | | | |

| **Supplemental Table 7.** The comparison of the completion time (seconds) of the mPG-SGA and the PG-SGA. | | | | | | | | |
| --- | --- | --- | --- | --- | --- | --- | --- | --- |
|  | **N** | **Mean (standard deviation)** | **Minimum** | **Maximum** | **Median** | **25th percentile** | **75th percentile** |  |
| **mPG-SGA** | 30 | 281 (59) | 160 | 410 | 283 | 234 | 320 |  |
| **PG-SGA** | 30 | 411 (77) | 300 | 591 | 404 | 358.25 | 449 |  |

**Supplemental Table 8.** Area under the receiver operating characteristic curve, and the sensitivity, specificity and agreement between the mPG-SGA and the PG-SGA (well-nourished or mildly, moderately, and severely malnourished)*

| **Tumour** | | **n** | **Well-nourished or mildly malnourished (PG-SGA score 0-3 points or more)†** | | | | **Moderately or severely malnourished (PG-SGA score 4-8 points or more)†** | | | | **Weighted kappa*** |
| --- | --- | --- | --- | --- | --- | --- | --- | --- | --- | --- | --- |
|  |  |  | **AUC** | **Cut-off** | **Sensitivity** | **Specificity** | **AUC** | **Cut-off** | **Sensitivity** | **Specificity** |  |
| Overall score | | 34,071 | 0.989 | 2.5 | 0.918 | 1.000 | 0.985 | 6.5 | 0.945 | 0.938 | 0.907 |
| Primary tumour location | | | |  |  |  |  |  |  |  |  |
|  | Pancreatic cancer | 468 | 0.993 | 2.5 | 0.963 | 1.000 | 0.986 | 6.5 | 0.955 | 0.918 | 0.909 |
|  | Biliary cancer | 121 | 0.994 | 2.5 | 0.948 | 1.000 | 0.985 | 6.5 | 0.918 | 1.000 | 0.902 |
|  | Oesophageal cancer | 2512 | 0.992 | 2.5 | 0.945 | 1.000 | 0.985 | 6.5 | 0.952 | 0.924 | 0.886 |
|  | Gastric cancer | 4517 | 0.990 | 2.5 | 0.934 | 1.000 | 0.984 | 6.5 | 0.950 | 0.923 | 0.883 |
|  | GIST | 45 | 0.974 | 2.5 | 0.933 | 1.000 | 1.000 | 6.5 | 1.000 | 1.000 | 0.945 |
|  | Colorectal cancer | 6686 | 0.989 | 2.5 | 0.926 | 1.000 | 0.984 | 6.5 | 0.939 | 0.934 | 0.898 |
|  | Liver cancer | 1315 | 0.989 | 2.5 | 0.925 | 1.000 | 0.984 | 6.5 | 0.945 | 0.901 | 0.900 |
|  | Brain cancer | 342 | 0.990 | 2.5 | 0.925 | 1.000 | 0.992 | 6.5 | 0.988 | 0.914 | 0.902 |
|  | Leukaemia | 871 | 0.983 | 2.5 | 0.908 | 1.000 | 0.958 | 6.5 | 0.878 | 0.949 | 0.854 |
|  | Lung cancer | 6913 | 0.987 | 2.5 | 0.900 | 1.000 | 0.988 | 6.5 | 0.944 | 0.955 | 0.902 |
|  | Ovarian cancer | 778 | 0.986 | 2.5 | 0.920 | 1.000 | 0.987 | 6.5 | 0.941 | 0.960 | 0.916 |
|  | Malignant lymphoma | 1009 | 0.978 | 2.5 | 0.887 | 1.000 | 0.980 | 6.5 | 0.926 | 0.946 | 0.887 |
|  | Cervical cancer | 1434 | 0.995 | 2.5 | 0.935 | 1.000 | 0.985 | 6.5 | 0.962 | 0.946 | 0.946 |
|  | Endometrial cancer | 404 | 0.993 | 2.5 | 0.880 | 1.000 | 0.990 | 6.5 | 0.949 | 0.947 | 0.912 |
|  | Prostate cancer | 298 | 0.986 | 2.5 | 0.883 | 1.000 | 0.982 | 6.5 | 0.947 | 0.958 | 0.911 |
|  | Bladder cancer | 270 | 0.982 | 2.5 | 0.875 | 1.000 | 1.000 | 6.5 | 1.000 | 0.984 | 0.910 |
|  | Nasopharyngeal carcinoma | 2308 | 0.991 | 2.5 | 0.935 | 1.000 | 0.986 | 6.5 | 0.942 | 0.935 | 0.936 |
|  | Breast cancer | 3687 | 0.982 | 2.5 | 0.871 | 1.000 | 0.988 | 6.5 | 0.941 | 0.947 | 0.895 |
|  | Other cancer | 1022 | 0.987 | 2.5 | 0.922 | 1.000 | 0.982 | 6.5 | 0.938 | 0.937 | 0.906 |

Abbreviations: PG-SGA, Patient-Generated Subjective Global Assessment; CI, confidence interval; AUC, area under the curve

*To show the results concisely, patients with well-nourished status and mild malnutrition status were combined into one group for calculation and presentation.

†Overall accuracy was calculated by the receiver operating characteristic area under the curve (ROC-AUC).

|  | **mPG-SGA** | | | | | | | | ***P**** |
| --- | --- | --- | --- | --- | --- | --- | --- | --- | --- |
|  | **Well-nourished or mildly malnourished** | |  | **Moderately malnourished** | |  | **Severely malnourished** | |  |
|  | **n** | **Mean (SD)** |  | **n** | **Mean (SD)** |  | **n** | **Mean (SD)** |  |
| Total PG-SGA score | 15853 | 2.1 (1.1) |  | 9259 | 6.2 (1.5) |  | 8959 | 12.5 (3.6) | < 0.001 |
| NRS 2002 score | 15853 | 1.2 (1.0) |  | 9259 | 2.0 (1.5) |  | 8959 | 2.9 (1.6) | < 0.001 |
| KPS | 15851 | 90.6 (8.3) |  | 9259 | 86.1 (12.0) |  | 8959 | 78.3 (16.8) | < 0.001 |
| Albumin | 15853 | 40.9 (4.9) |  | 9259 | 39.1 (5.3) |  | 8959 | 37.0 (5.8) | < 0.001 |
| Total protein | 15778 | 70.1 (76.5) |  | 9201 | 67.5 (8.5) |  | 8922 | 67.4 (94.5) | < 0.001 |
| C-reactive protein | 4517 | 11.9 (35.9) |  | 2784 | 19.0 (33.3) |  | 2879 | 30.4 (44.2) | < 0.001 |
| Haemoglobin | 15853 | 126.1 (20.8) |  | 9259 | 121.3 (22.0) |  | 8959 | 115.1 (23.1) | < 0.001 |
| BMI | 15853 | 23.4 (3.3) |  | 9259 | 22.5 (3.4) |  | 8959 | 21.2 (3.4) | < 0.001 |
| Upper midpoint arm circumference | 14377 | 27.2 (25.3) |  | 8308 | 26.3 (5.4) |  | 7984 | 25.0 (4.7) | < 0.001 |
| Triceps skin fold thickness | 14311 | 17.7 (8.9) |  | 8268 | 15.7 (8.7) |  | 7954 | 13.6 (7.6) | < 0.001 |
| Non-sharp (or non-injury) grip | 14061 | 26.3 (40.5) |  | 8044 | 25.1 (20.1) |  | 7630 | 22.3 (10.9) | < 0.001 |

**Supplemental Table 9.** Characteristics of the patients based on the nutritional assessment according to the mPG-SGA (N=34071)

*Derived from ANOVA for continuous variables.


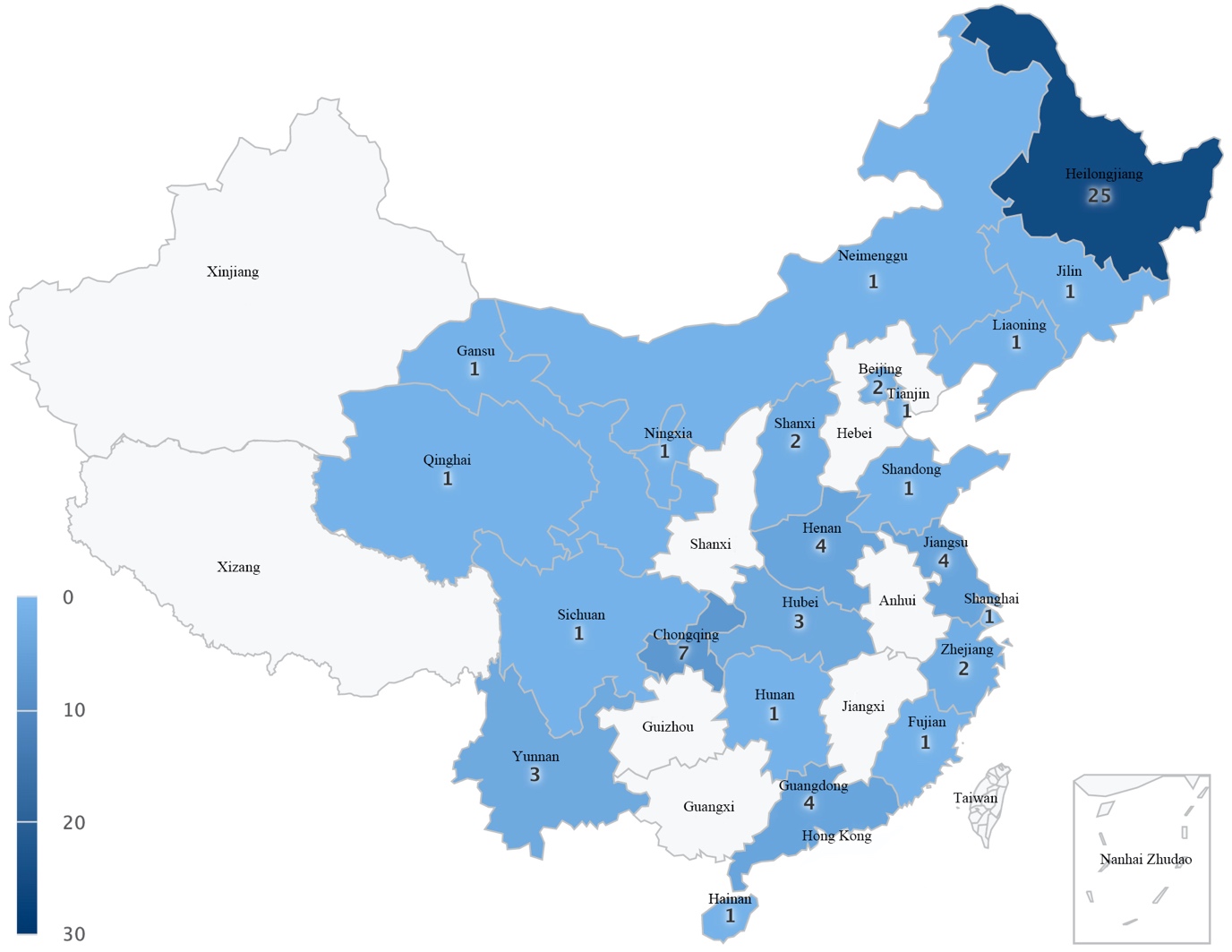


**Supplemental Figure 1.** The locations where the professionals worked
